# Great debate: device-detected subclinical atrial fibrillation should be treated like clinical atrial fibrillation

**DOI:** 10.1093/eurheartj/ehae365

**Published:** 2024-06-27

**Authors:** Prashanthan Sanders, Emma Svennberg, Søren Z Diederichsen, Harry J G M Crijns, Pier D Lambiase, Giuseppe Boriani, Isabelle C Van Gelder

**Affiliations:** Centre for Heart Rhythm Disorders, University of Adelaide and Royal Adelaide Hospital, Port Road, 5000 Adelaide, Australia; Karolinska Institutet, Department of Medicine, Huddinge, Karolinska University Hospital, Stockholm, Sweden; Department of Cardiology, Copenhagen University Hospital—Rigshospitalet, Copenhagen, Denmark; Department of Cardiology and Cardiovascular Research Centre Maastricht (CARIM), Maastricht University Medical Centre, Maastricht, The Netherlands; Cardiology, University College London & Barts Heart Centre, London, UK; Cardiology Division, Department of Biomedical, Metabolic and Neural Sciences, University of Modena and Reggio Emilia, Policlinico di Modena, Modena, Italy; Department of Cardiology, University of Groningen, University Medical Centre Groningen, Groningen, The Netherlands

**Keywords:** Subclinical atrial fibrillation, Device-detected atrial fibrillation, Atrial fibrillation burden, Oral anticoagulation

## Abstract

**Graphical Abstract:**
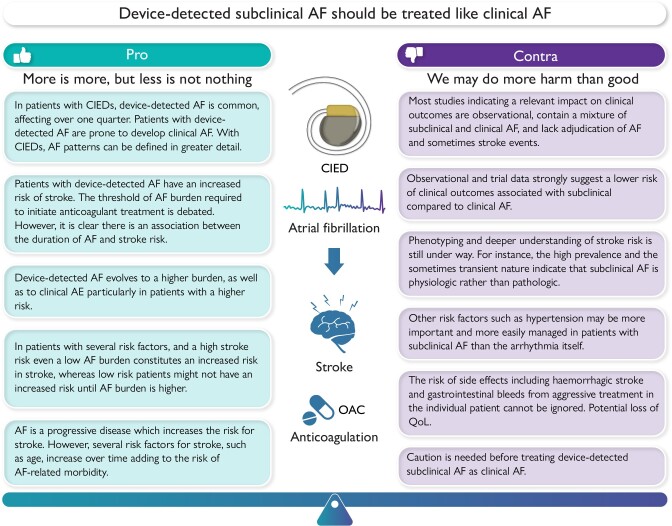
Summary of the factors representing the equipoise associated with device-detected subclinical atrial fibrillation to inform patient-specific treatment. AF, atrial fibrillation; CIED, cardiac implantable electronic device; OAC, oral anticoagulation; QoL, quality of life.

Summary of the factors representing the equipoise associated with device-detected subclinical atrial fibrillation to inform patient-specific treatment. AF, atrial fibrillation; CIED, cardiac implantable electronic device; OAC, oral anticoagulation; QoL, quality of life.

## Introduction

https://orcid.org/0000-0003-3803-8429SandersPrashanthan
Centre for Heart Rhythm Disorders, University of Adelaide and Royal Adelaide Hospital, Adelaide, Australia

Automated continuous rhythm monitoring has been made possible with the extensive use of cardiac implantable electronic devices (CIEDs). A highly prevalent finding in devices with atrial sensing capabilities is atrial high-rate episodes (AHREs). These events have had variable definitions in the literature but are commonly referred to as atrial tachyarrhythmias with a rate of >175 b.p.m. and a duration of >5 min.^1–3^ False positives are fairly common—contributed by artefacts, noise, and far-field R-waves.^4^ Subclinical atrial fibrillation (SCAF) describes mostly asymptomatic device-detected AHREs confirmed to be AF, atrial flutter, or atrial tachycardia after a visual review of CIED tracings. In contrast, clinical AF is defined as symptomatic or asymptomatic AF of at least 30 s and documented by surface electrocardiogram (ECG).^5^ A recent meta-analysis of 54 studies reported a pooled prevalence of SCAF of 28.1% and found SCAF to be more frequent in older patients with multiple comorbidities and higher thromboembolic risks.^6^ At present, there is a knowledge gap regarding the clinical significance of short episodes of SCAF and the optimal management approach especially surrounding oral anticoagulation (OAC) therapy.

Currently, available evidence shows mixed results delineating the association between SCAF and its risk of stroke. TRENDS and MOST studies showed an increased risk of stroke, along with cardiovascular and all-cause mortality in patients with SCAF, and their findings were supported by other large trials.^1–3,7^ An important randomized trial, the STROKESTOP study, highlighted the benefit of treating asymptomatic AF towards reducing the primary combined endpoint of ischaemic stroke, systemic embolism, death from any cause, haemorrhagic stroke, or hospitalization for bleeding.^8^ However, previous studies have shown that, although stroke risk for patients with SCAF is higher than those without, it is lower than for patients with clinical AF.^9–12^ The LOOP study also had negative results with no significant reduction in the primary endpoint despite higher detection of AF from loop recorders with subsequent increase in the initiation of OAC.^13^

It has been postulated that the risk of stroke is related to the duration and burden of SCAF. Longer duration of SCAF episodes between 12 and 23 h is independently associated with the risk of clinical AF.^14^ Device-detected AF and higher CHA_2_DS_2_-VASc scores at baseline also predict the subsequent development of clinical AF.^15^ These factors possibly contributed to the higher risk of stroke with SCAF episodes lasting >24 h as reported in the ASSERT study.^9^ The heterogeneity of SCAF definition and differences in previously analysed patient populations add to the complexity of determining the optimal burden and duration of SCAF that could benefit from OAC therapy.

The risk of bleeding is also a major concern, as studies of novel OAC have demonstrated a risk of bleeding from 3% to 5%.^16–20^ Two large randomized controlled trials, NOAH-AFNET 6 and ARTESIA, have recently added to our understanding of the role of nonvitamin K antagonist OAC in this group of patients.^21,22^ Although providing further information, the management of device-detected silent AF should be focused on offering a personalized, patient-centred approach; balancing the risks and benefits of OAC initiation; as well as identifying and reducing the factors driving AF progression to improve overall clinical outcomes.

Although providing the clinician with greater granularity of rhythm monitoring, several important questions remain. Here, we present the debate on the equipoise related to device-detected SCAF and how the clinician should manage these patients (*Graphical Abstract*).

## Figures and Tables

**Figure 1:**
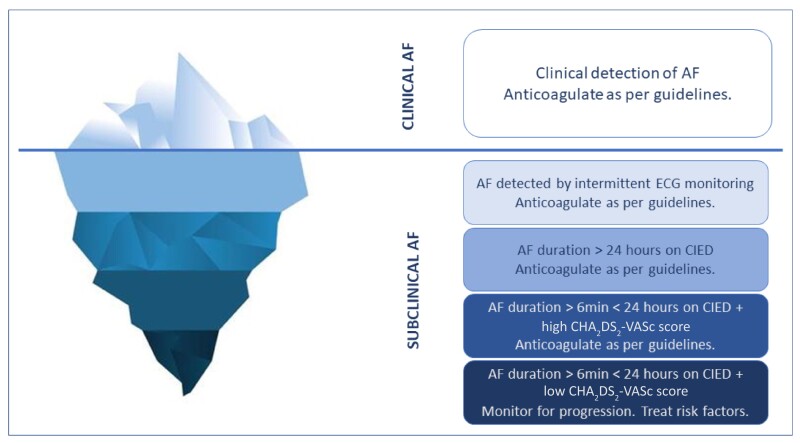
The enormity of the burden of subclinical atrial fibrillation and suggested clinical guidance for the management of anticoagulation in the various scenarios of subclinical atrial fibrillation burden. AF, atrial fibrillation; CIED, cardiac implantable electronic device; ECG, electrocardiogram

**Figure 2:**
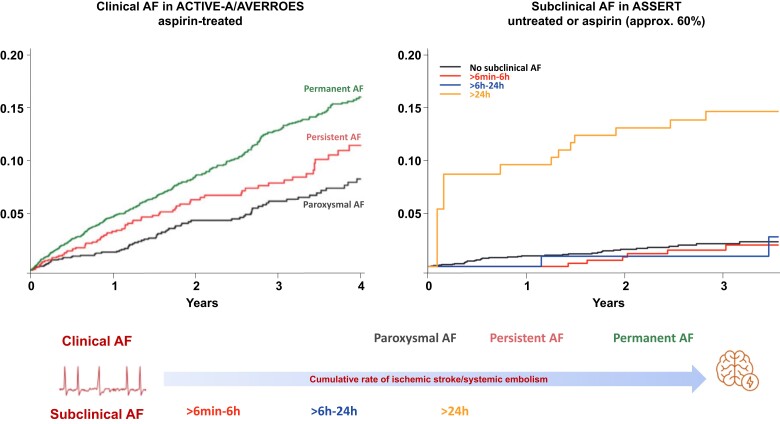
Cumulative rate of ischaemic stroke/systemic embolism in the absence of anticoagulation in clinical atrial fibrillation (left panel)^2^ compared with subclinical atrial fibrillation of varying durations (right panel).^3^ SCAF, subclinical atrial fibrillation

## Pro

https://orcid.org/0000-0002-6413-0870SvennbergEmma
Karolinska Institutet, Department of Medicine, Huddinge, Karolinska University Hospital, Stockholm, Swedenhttps://orcid.org/0000-0003-1073-5337CrijnsHarry J G M
Department of Cardiology and Cardiovascular Research Centre Maastricht (CARIM), Maastricht University Medical Centre, Maastricht, The Netherlandshttps://orcid.org/0000-0002-9820-4815BorianiGiuseppe
Cardiology Division, Department of Metabolic and Neural Sciences, University of Modena and Reggio Emilia, Policlinico di Modena, Modena, Italy

Corresponding author: Emma Svennberg.

Most medical risks in medicine are not dichotomous, but rather exist on a continuous scale. One common clinical example is hypertension, where patients with well-controlled hypertension have a substantially lower risk compared with a patient with grade 3 hypertension. Although grade 3 hypertension is associated with increased risk, milder forms of hypertension also have worse outcomes than normal blood pressure, and treatment should be initiated and continued life-long for most of these patients.^1^ The same might also be true for atrial fibrillation (AF), and as in hypertension, milder forms can progress over time or, in combination with other risk factors, become more malignant.

AF was initially described as a binary disease, likely due to low monitoring resources with subsequent difficulties in detecting AF that was not permanent or persistent. Over 30 years ago, it was discovered that patients found to have a dichotomous AF diagnosis had a five-fold increased risk of stroke.^2^ When increasing monitoring capabilities patterns of AF emerged, a categorical division was employed, still used in current European Society of Cardiology guidelines for AF, where AF is described depending on its temporal pattern as paroxysmal, persistent, long-standing persistent, or permanent.^3^ Although these intra-individual patterns of AF vary substantially,^4^ many studies have used this division to describe AF, and, despite its drawbacks, AF patterns are associated with clinical outcomes. Patients with paroxysmal AF have been shown to have a lower risk of stroke and systemic embolism compared with nonparoxysmal patients, yet, in the trials, a consistent reduction in outcomes was seen regardless of AF pattern.^5^ AF patterns form the basis for many treatment decisions, in particular for rhythm control, but does not guide treatment with oral anticoagulation (OAC) therapy.

The term subclinical AF (SCAF) implies a lack of symptoms or symptoms diffuse enough not to have been brought to clinical attention. One could potentially think that a lack of symptoms is synonymous with a lower risk of adverse outcomes. This notion has, however, been dispelled in trials, where outcomes in AF patients showed no difference with regard to stroke or all-cause mortality regardless of symptomatology.^6^ The new term ‘device-detected’ AF has been suggested to replace ‘subclinical’ to better describe the entity.^7^

Two recently published large randomized controlled studies have used different modalities to determine if screening for asymptomatic AF can prevent AF-associated morbidity. In the pragmatic STROKESTOP study, >28 000 individuals aged 75–76 years residing in 2 Swedish regions were randomized to be screened for AF using intermittent handheld 30 s electrocardiogram (ECG) recordings twice daily for 2 weeks. Although only about half of the invitees chose to participate, the intention-to-treat analysis showed a small benefit in favour of screening with regard to the primary combined endpoint of stroke, death, and severe bleeding after a minimum follow-up of 5.5 years.^8^

In the LOOP study, a different screening approach was used. After an initial screening visit, 6004 individuals aged 70–90 years with 1 additional risk factor were randomized 1:3 to receive an implantable loop recorder or standard care. The study was powered to detect a difference of 35% between the groups with regard to their combined endpoint of stroke, transient ischaemic attack, and systemic embolism. Although AF detection over 6 min was common and led to initiation of OAC therapy, the study failed to reach its highly set target in its intention to treat analysis but showed a benefit in favour of screening in the per protocol analysis.^9^

It is likely that individuals that have AF detected using a method that monitors the heart rhythm for such a short monitoring period (intermittent ECG recordings twice daily for 2 weeks) compared with continuous monitoring represent patients with a high burden of AF^10^ that might also have a higher risk of downstream negative consequences of AF. So, this type of SCAF, i.e. detected during monitoring twice daily for a fortnight, should be treated like clinical AF. A meta-analysis of randomized controlled trials showed that AF screening was associated with a reduction in stroke [relative risk (RR) 0.91; 95% confidence interval (CI) 0.84–0.99], but suggested that more studies were needed.^11^ The ongoing SAFER trial (https://www.safer.phpc.cam.ac.uk) will contribute more data.

For patients with cardiac implantable electronic devices (CIEDs), AF patterns can be defined in greater detail, mainly by measuring how much time the patient is in AF divided by the monitoring time, commonly known as AF burden^12^ or the duration of the longest AF episode. In CIED carriers, device-detected AF is common, affecting over one quarter of monitored patients.^13^ Atrial fibrillation is a progressive disease, and the duration and pattern of AF change over the patient's life.^14^ In a study combining patient data from three CIED studies, it was shown that among the 2244 patients with a SCAF burden of ≥5 min, almost half (49.8%) progressed to a higher AF burden over time, and almost one quarter (24%) transitioned to a burden of ≥23 h during follow-up.^15^ Patients with SCAF are also prone to develop clinical AF.^13,15,16^

In the landmark ASSERT study, episodes of atrial arrhythmias >6 min in CIED carriers were shown to be associated with a 2.5-fold increased risk of stroke.^17^ Similar risks have been shown in a recent meta-analysis encompassing 4961 patients across 7 studies with atrial high-rate episodes (AHREs; importantly free of clinical AF). This showed similar risks of thromboembolic events (risk ratio 2.13), but with a large individual variation as reflected in the 95% CI of 1.53–2.95.^16^ Although this risk is lower than the commonly quoted five-fold increased risk for stroke in AF patients, as reported three decades ago, it still remains clearly elevated.^2^

Although individual differences are vast, and it is well known that the risk for AF-related outcomes increases with comorbidities, the focus of recent debates has been on the duration of the arrhythmia episodes. In particular, a lot of emphasis has been paid to the SCAF episode duration required to increase stroke risk with variable results, ranging from an increased risk for stroke in AHRE >5 min^18^ to >5.5 h^19^ or requiring >24 h^20^ leading to uncertainty in clinical practice with subsequent variations in OAC prescription for these patients.

Recently, two large studies, the NOAH-AFNET 6^21^ and the ARTESIA^22^ trials, randomized patients with an increased risk of stroke to either receive an OAC or to a control group. In the ARTESIA trial, the comparator drug was aspirin, and in the NOAH-AFNET 6 trial, it was no treatment or, if clinically indicated, an antiplatelet drug. As seen in observational trials, the risk of stroke in patients with device-detected AF was lower compared with clinical stroke, with annual rates of ischaemic stroke in control groups of 1.1% and 1.0% in NOAH-AFNET-6 and ARTESIA, respectively. In the larger ARTESIA trial, 4012 individuals, with a mean age of 77 years and an average CHA_2_DS_2_-VASc score of 3.9, were followed for a mean of 3.5 years. In the intention-to-treat analysis, it was shown that treatment with apixaban reduced the risk of the primary endpoint of stroke and systemic embolism by 37% [hazard ratio (HR) 0.63; 95% CI 0.45–0.88; *P* = 0.007]. In particular, the risk of fatal or disabling strokes, as classified by the modified Rankin scale, was reduced by more than half in the apixaban group compared with the group treated with aspirin (HR 0.51; 95% CI 0.29–0.88).^22^

In comparison, the NOAH-AFNET 6 trial that included 2536 individuals, with a mean age of 78 years and an average CHA_2_DS_2_-VASc score of 4, was terminated early due to futility and risk of harm after 21 months. The primary endpoint of the NOAH-AFNET 6 trial, of cardiovascular death, stroke, and systemic embolism, was not met.^21^

In both trials, the risk of bleeding in the group randomized to OAC treatment was increased compared with the comparator group; in ARTESIA, the risk of bleeding was increased by 36% (HR 1.36; 95% CI 1.01–1.82) in the apixaban group compared with the aspirin group in the intention-to-treat analysis, and similarly in the NOAH-AFNET 6 trial, the group randomized to edoxaban had an increased risk of bleeding (HR 2.10; 95% CI 1.30–3.38).^23^

In a meta-analysis of the two trials, it was shown, with a high degree of consistency, that there was a significant reduction in the endpoint of ischaemic stroke with OAC (RR 0.68, 95% CI 0.5–0.92, *I*^2^ = 0%), as well as in the combined endpoint of ischaemic stroke or systemic embolism (RR 0.63, 95% CI 0.47–0.84, *I*^2^ = 0%).^24^ No difference was seen between the groups with regard to cardiovascular mortality, nor all-cause mortality. In the meta-analysis, it was shown that the risk of bleeding on OAC was increased compared with the control group (RR 1.62, 95% CI 1.05–2.5, *I*^2^ = 61%), but with no increase in fatal bleeding (RR 0.79, 95% CI 0.37–1.69, *I*^2^ = 0%).

In both trials, progression to clinical AF was common. In the ARTESIA trial patients, only individuals with device-detected AF <24 h were included, and in cases where AF progressed to >24 h or clinical AF was detected, patients were censored from the trial, and OAC treatment commenced (24% of all patients). In the NOAH-AFNET 6 trial, there was no restriction on the duration of the device-detected episode, but patients with clinical AF detected were similarly censored from the trial (18.2% of all patients, 8.7% per patient-year). In a sub-analysis of the NOAH-AFNET 6 trials, it has been shown that progression to clinical AF is more common in individuals with durations of device-detected AF exceeding 24 h.^25^

Patients with clinical AF should be treated with OAC if they have an increased risk of stroke (*Figure 1*).^3^ It is becoming increasingly clear that also patients with device-detected AF should be treated with OAC, as this will reduce the risk of severe strokes, albeit at an increased risk of nonfatal bleeding.^24^ Certainly, patients need to be informed about the increased risk of bleeding, in particular gastrointestinal bleeding, and a close eye be kept on bleeding symptoms and haemoglobin levels.

Early AF has in one randomized controlled trial been shown to respond particularly well to treatment with rhythm control.^26^ Rhythm management in SCAF patients has not been prospectively tested.

Most patients with AF have several cardiovascular comorbidities that increase the risk of stroke in AF.^3^ It is not only the duration of the longest AF episode (or AF burden) that matters but also a more complex interplay of the patients’ comorbidities and AF duration.^27^ More recent studies have nicely depicted this interplay between the stroke risk factors and AF burden.^28^ In patients with several risk factors and a high stroke risk, even a low AF burden constitutes an increased risk in stroke.^22,29^ Re-evaluation of these patients is also of essence, as not only the progressive nature of AF itself increases the risk for stroke but also risk factors for stroke, such as age, increase over time adding to the risk of AF-related morbidity.^30^

Queries with regard to the temporal association between SCAF and ischaemic stroke further raised the question if AHREs are a risk marker for atrial disease and other causes of stroke rather than the rhythm being a risk factor *per se* in itself,^31^ but studies have been hampered by poor adjudication of stroke subtype and few outcomes leading to uncertainty.^32,33^ In a more recent case–crossover trial, patients with ischaemic strokes preceded by at least 4 months of continuous heart rhythm monitoring were assessed. In the study, patients with a SCAF >5.5 h had a 3.7-fold increase in stroke risk, increasing to 5-fold in case of episodes >24 h. This risk was particularly high within 5 days of an AF episode.^34^ Similarly, in large cohort studies, a clear temporal relationship between stroke and AF emerged.^35^ The importance of temporality between AF and stroke is currently being prospectively tested in a trial using guided monitoring of AF episodes and pill-in-the-pocket OAC treatment.^36^

Over time, much attention in AF treatment has been on preventing ischaemic stroke, but one should bear in mind that heart failure is a more common, potentially lethal outcome in patients with AF.^3,37^ Progression of SCAF burden has been shown to increase the risk of heart failure hospitalization five-fold.^38^ Hence, to reduce the risk of progression, early rhythm management^26^ or rate management (if high burden) to reduce the risk of tachycardia-mediated cardiomyopathy should be considered in SCAF patients. In particular, when AF is associated with heart failure, SCAF should be carefully managed as reducing AF burden in heart failure patients is associated with a favourable prognosis.^39,40^

In conclusion, as we are moving away from the binary yes and no in the diagnosis of AF to a more continuous approach, this should lead to an amendment of the simplistic thinking with regard to risk and treatment of SCAF. SCAF episodes need to be seen in the context of the patient instead of focusing on the duration of a single episode. Most patients with SCAF should be treated as patients with clinical AF, with treatment of risk factors, and stroke preventive treatment in patients at high risk of stroke. For a few patients, the burden of AF and risk factor pattern do not yet merit further stroke preventive treatment. These patients should be carefully observed over time, as the risk of progression has been clearly shown. SCAF is also an opportunity to initiate treatment of comorbidities to prevent not only stroke but also progression of SCAF episodes and subsequent increased risk of heart failure.

In summary, we suggest following SCAF patients as clinical AF patients, with management focussing on comorbidities, emerging symptoms, perceived risks of heart failure and stroke (if unfavourable burden-modified CHA_2_DS_2_-VASc score), and risk of AF progression. Although not as easy as a binary approach, in the words of Sir William Osler, ‘If it were not for the great variability among individuals, Medicine might be a Science, not an Art’.

## Contra

https://orcid.org/0000-0002-9687-0857DiederichsenSøren Z
Department of Cardiology, Copenhagen University Hospital—Rigshospitalet, Copenhagen, Denmarkhttps://orcid.org/0000-0002-9055-9267LambiasePier D
Cardiology, University College London & Barts Heart Centre, London, UKhttps://orcid.org/0000-0002-7579-1201Van GelderIsabelle C
Department of Cardiology, University of Groningen, University Medical Centre Groningen, Groningen, The Netherlands

Corresponding author: Søren Z Diederichsen.

The last 20 years has seen a journey from the initial phenotyping of subclinical atrial fibrillation (AF) to defining its implications for clinical outcomes. In patients with cardiac implantable electronic devices (CIEDs), AF is defined as atrial high-rate episodes (AHREs) confirmed to be AF by a cardiologist as device-detected AF. Device-detected AF is most often asymptomatic but is associated both with progression to clinical AF [as defined by AF detected by standard electrocardiogram (ECG)] and with increased stroke risk. This has led to arguments that device-detected AF should be managed like clinical AF. Here, we offer arguments that it should not.

The most important argument adheres to the low stroke rate observed in patients with device-detected AF compared with clinical AF. The ASSERT trial was the first to investigate the association between adjudicated device-detected AF and stroke risk.^1^ This trial enrolled 2580 patients with a recent device implantation and without known AF. During a mean of 2.5 years of follow-up, 35% of patients had device-detected AF episodes lasting ≥6 min, and 2.0% suffered a stroke or systemic embolism (51 events total). Anticoagulation use was essentially zero. Device-detected AF during the first 3 months was associated with a stroke rate of 1.7% per year going forward. While this was higher than for patients without AF in the trial, it was not as high as the 2.1%–4.2% per year in nonanticoagulated patients with paroxysmal to permanent AF in the ACTIVE-A and AVERROES studies (*Figure 2*).^2^ A subsequent analysis found that the increased stroke risk was upheld by patients with episodes lasting ≥24 h, while the risk associated with shorter episodes was comparable with that of no AF at all.^3^ The low stroke rate in patients with short device-detected AF episodes was also noted in a large registry-based study merging health records with a CIED database.^4^

At the initial reporting of ASSERT, the LOOP study was designed to assess whether detection and treatment of subclinical AF would decrease stroke risk in patients without CIED but with risk factors likely to yield a benefit from such intervention.^5^ The trial randomized 6004 participants to implantable loop recorder (ILR) screening and anticoagulation initiation upon detection of AF episodes lasting ≥6 min vs. usual care. Even in this CIED-naïve population, 30% of those screened had subclinical AF and anticoagulation was initiated in 93% of these.^6^ Stroke occurrence was higher than in ASSERT with a total of 318 patients (5.3%) suffering a primary outcome of stroke or systemic embolism (315 and 3 events, respectively) and an event rate of 1.1% per year in the control group. The result was neutral though there was a trend towards decreased risk in the screening group [hazard ratio (HR) 0.80, 95% confidence interval (CI) 0.61–1.05].^6^

Recently, two double-blind, double-dummy, randomized trials were reported looking specifically at anticoagulation after detection of subclinical AF.^7,8^ NOAH-AFNET 6 randomized 2536 patients with CIED-detected subclinical AF episodes lasting ≥6 min and additional risk factors to edoxaban vs. placebo (or aspirin if indicated at baseline, which was the case for 54%).^8^ The trial was stopped when it was deemed futile for demonstration of efficacy for the primary outcome of stroke, systemic embolism, or cardiovascular death (HR 0.81, 95% CI 0.60–1.08) but revealed a significant increase in major bleeding (HR 1.31, 95% CI 1.02–1.67). The rate of ischaemic stroke was low at 1.1% per year in the placebo group, and there was no convincing signal towards the benefit for this outcome (HR 0.79, 95% CI 0.45–1.39). A sub-analysis found equally low rates of ischaemic stroke among the 259 patients (11%) with AF episodes lasting ≥24 h at baseline [2 strokes among 127 patients in the placebo group (0.97%/year) and 2 among 132 patients in the edoxaban group (0.95%/year)].^9^ ARTESIA randomized 4012 patients with CIED-detected subclinical AF episodes lasting between 6 and 24 h to apixaban vs. aspirin, finding that apixaban was associated with a decrease in the primary outcome of stroke and systemic embolism (HR 0.63, 95% CI 0.45–0.88), but again with an increase in major bleedings (HR 1.80, 95% CI 1.26–2.57).^7^ The number needed to treat to prevent 1 primary outcome was 250 compared with a number needed to harm of 130. As with NOAH-AFNET 6, the rate of ischaemic stroke was low at only 1.0% per year in the comparator arm.

The main question nowadays seems to be whether it is AF itself or the associated comorbidities that set the stage for stroke risk. In other words, is AF the mechanism or is it just a bystander? To elucidate answers, we need to better understand the natural histories of AF, stroke, and associated risk factors. Proponents of early treatment would argue that short, device-detected AF is a precursor for clinical AF and even heart failure,^10,11^ but while this may be true for some cases, there are many where AF resolves spontaneously, as seen in up to approximately 25%–50% of cases in the LOOP study.^12^ The extremely high prevalence of approximately 30% in various populations^1,6,13–16^ may itself pose an argument that device-detected subclinical AF should merely be considered part of normal physiology and ageing. Even if device-detected subclinical AF is indeed a precursor for clinical AF, questions remain as to whether clinical action can await clinical diagnosis, e.g. due to appearance of longer episodes or symptoms.

Another issue relates to the lack of temporal relationship, if any relationship, between AF and stroke in many cases. Only 4 (8%) of the above-mentioned 51 strokes in ASSERT were preceded by AF,^17^ and AF was only detected in 17 of 67 (25%) ILR participants suffering a stroke in LOOP—a proportion that was lower than for all ILR participants with or without stroke.^6^ Lacking temporal patterns have also been reported in older studies such as TRENDS^18^ and IMPACT.^19^ As such, the majority of strokes seem to occur in patients with zero AF, and most of the remaining strokes cannot be linked to AF despite continuous monitoring. Even though a temporal relationship may exist in some cases, many strokes seem to occur with no AF, with few of the remaining patients having AF in the month preceding the event.^20^

Furthermore, we may consider AF burden as a relevant target rather than binary AF ‘present vs. absent’, which could also pose an argument against the treatment of device-detected subclinical AF *per se*. Observational studies have suggested that higher AF burden is associated with higher stroke risk, having looked at burden as defined by percentage of time,^21^ longest episode duration,^3^ or even using a combination of episode duration and comorbidities.^4^ In general, low AF burden seems to be associated with low risk, often comparable to zero AF. It should be noted that clear definitions and clinically relevant cut-offs for AF burden are still lacking, and any definition would have limitations with regard to how AF was captured and how often re-assessments should be made, considering the various temporal patterns of AF.

The higher stroke rates observed with higher AF burden may underlie the signal towards preventive effect of screening in the only positive AF screening trial, namely, STROKESTOP. This was a population-based trial including 28 768 persons randomized to invitation to screening using handheld single-lead 30 s ECGs twice daily for 2 weeks vs. usual care. Approximately half of those invited attended the screening. The result was positive for the combined outcome of stroke, systemic embolism, bleeding, and all-cause death for invitation to screening compared with usual care, although with a small effect size (HR 0.96, 95% CI 0.92–1.00) with the difference occurring very late during follow-up and with no difference in anticoagulation treatment.^22^ Looking in more detail, the screening yield was only 1.4% per protocol (3.0% by intention to screen), and the majority of AF diagnoses were made during the first days.^23^ This indicates that the diagnoses made here were of a different AF phenotype than what is usually detected by CIED and inferences should not be made from STROKESTOP on how to treat device-detected subclinical AF.

Much of our understanding of device-detected atrial tachyarrhythmias comes from studies with important limitations. Although being pivotal in their description of the association between device-detected subclinical AF or AHREs and stroke, neither the aforementioned TRENDS study^24^ nor an ancillary analysis of the MOST trial^25^ excluded patients with clinical AF or adjudicated CIED annotations to be subclinical AF as opposed to e.g. electrical noise or other arrhythmia. In TRENDS, device-detected subclinical AF occurred in 47%, while 20% had previously documented clinical AF,^24^ and while AF lasting >5 min was detected in 51% of patients in the MOST analysis, an even higher proportion had atrial tachyarrhythmias at baseline (60%; 81% and 39% for patients with and without AHREs detected, respectively).^26^ Other studies have similar limitations.^27,28^ As such, it seems reasonable to question to which extent the older observations of high stroke risk were upheld by prevalent clinical arrhythmias and associated risk factors as opposed to subclinical AF alone. This leads us to the concept of atrial cardiomyopathies.^29,30^

Even though several trials demonstrated a very high yield of AF detection using extended monitoring after embolic stroke of undetermined source (ESUS) or transient ischaemic attack,^31–34^ this did not translate into all patients with ESUS benefitting from anticoagulation.^35,36^ A *post hoc* analysis of one of the ESUS trials found that anticoagulation was associated with reduced risk of recurrent stroke in patients with left atrial enlargement and no documented AF.^37^ Such findings, along with other observations of association between atrial markers and stroke risk independent of AF, have sparked an interest in atrial cardiomyopathy as a target rather than the arrhythmia itself,^38^ though definitions and net benefit of treatment are not yet established.

In line with this, we offer a last argument that any efforts directed towards device-detected subclinical AF should not get in the way of caring for other, more well-described risk factors. *Post hoc* analyses of LOOP found no signal towards benefit of AF screening and treatment in patients with established heart disease^39^ or even prior stroke^40^ despite these patients having higher event rates overall. Also, only a minority of ischaemic strokes (14%) were deemed cardioembolic.^40^ These findings highlight the problem with competing risk factors, i.e. that interventions targeted towards a specific condition may be ineffective in the presence of others. Finally, the risk of side effects from aggressive anticoagulation treatment cannot be ignored, as there are worrisome signals of major bleeding in NOAH-AFNET 6, ARTESIA, and ESUS trials, where individual patient's bleeding risk doubled after OAC initiation compared with pre-treatment in LOOP.^7,8,35,36,41^ Before focusing on glimpses of asymptomatic device-detected AF, some patients may warrant good care of other, potentially more manageable conditions, such as hypertension,^42,43^ before focus is directed towards anticoagulation. A focus on cardiovascular and comorbidity optimization as part of the holistic pathway for the management of AF is warranted.^44^

To summarize, we should not treat device-detected subclinical AF as clinical AF for the following reasons: (i) device-detected subclinical AF is a common and often nonprogressive condition that may be considered part of normal physiology and ageing; (ii) device-detected subclinical AF is associated with a benign pathophysiology and lower stroke risk compared with clinical AF, especially when AF burden is low; (iii) randomized studies have not convincingly demonstrated net benefit for anticoagulation of device-detected subclinical AF, especially considering risk of bleeding from anticoagulation; and (iv) clear treatment targets have yet to be defined and any focus on device-detected subclinical AF should not steal focus from other risk factors that may be more important than the arrhythmia itself. In short, we should be careful not to treat device-detected subclinical AF as clinical AF, because the evidence is lacking and we may do more harm than good.
